# Musculoskeletal pitfalls in ^68^Ga-PSMA PET/CT

**DOI:** 10.1590/0100-3984.2023.0003

**Published:** 2023

**Authors:** Írline Cordeiro de Macedo Pontes, Anthony Reis Souza, Eduardo Kaiser Ururahy Nunes Fonseca, Akemi Osawa, Ronaldo Hueb Baroni, Adham do Amaral e Castro

**Affiliations:** 1 Imaging Department, Hospital Israelita Albert Einstein, São Paulo, SP, Brazil

**Keywords:** Gallium radioisotopes/metabolism, Prostate-specific antigen/metabolism, Positron emission tomography computed tomography, Musculoskeletal diseases/diagnostic imaging, Radioisótopos de gálio/metabolismo, Antígeno prostático específico/metabolismo, Tomografia por emissão de pósitrons combinada a tomografia computadorizada, Doenças musculoesqueléticas/diagnóstico por imagem

## Abstract

Prostate-specific membrane antigen (PSMA) is a transmembrane protein expressed in normal prostate cells and overexpressed in prostate cancer. Consequently, it is an important tool in the evaluation of prostate cancer, including the staging of high-risk patients and the assessment of biochemical recurrence. Despite the “specific” designation, benign musculoskeletal conditions, such as fractures, osteodegenerative changes, and fibrous dysplasia, can also show PSMA uptake, which can lead to misinterpretation of the imaging findings. Therefore, radiologists must be aware of these potential pitfalls, understand their causes, and fully analyze their morphologic features on unfused computed tomography (CT) and magnetic resonance imaging scans to correctly interpret the examination. In this pictorial essay, we review the basic characteristics of the ^68^Ga-PSMA positron-emission tomography/CT (PET/CT) radiotracer, discuss potential causes of false-positive findings on ^68^Ga-PSMA PET/CT in the musculoskeletal system, and illustrate the corresponding imaging findings.

## Abstract (pt)

O antígeno de membrana próstata específico (PSMA) é uma proteína transmembrana que apresenta expressão em células prostáticas normais e superexpressão em neoplasia da próstata. Dessa forma, é uma importante ferramenta na avaliação da neoplasia prostática, de utilidade no estadiamento de pacientes de alto risco e na análise de recorrência bioquímica. Apesar do termo “específico”, condições musculoesqueléticas benignas podem demonstrar captação de PSMA, como fraturas, alterações osteodegenerativas e displasia fibrosa, podendo levar a uma avaliação equivocada dos achados de imagem. Assim, o radiologista deve conhecer esses potenciais *pitfalls*, compreender suas causas e analisar as características morfológicas nas imagens não fundidas de TC e RM para interpretar corretamente o exame. Neste ensaio iconográfico, revisaremos as características básicas do radiofármaco ^68^Ga-PSMA PET/CT, discutiremos possíveis causas de resultados falso-positivos na ^68^Ga-PSMA PET/CT no sistema musculoesquelético e ilustraremos os achados de imagem correspondentes.

## INTRODUCTION

Prostate-specific membrane antigen (PSMA) is a transmembrane protein that is overexpressed in prostate cancer cells in comparison with benign prostatic tissue (more than 100 times greater expression). Consequently, its clinical application in prostate cancer has expanded rapidly, especially in staging high-risk patients and in evaluating biochemical recurrence^(1)^. Although called “specific”, PSMA is a folate hydrolase that is expressed in a variety of normal tissues, neovascularized tissues, and (benign and malignant) tumors other than those of the prostate^(2)^. Therefore, physicians need to be aware of and promptly recognize potential pitfalls related to PSMA uptake to avoid misinterpretation. In this pictorial essay, we aim to review the basic characteristics of the ^68^Ga-PSMA positron-emission tomography/computed tomography (PET/CT) radiotracer, to discuss potential causes of false-positive findings on ^68^Ga-PSMA PET/CT in the musculoskeletal system, and to illustrate the imaging findings, including a review of unfused CT images, for optimal skeletal evaluation when interpreting ^68^Ga-PSMA PET/CT findings.

## PSMA RADIOTRACER UPTAKE AND NORMAL DISTRIBUTION

**Figure 1:**
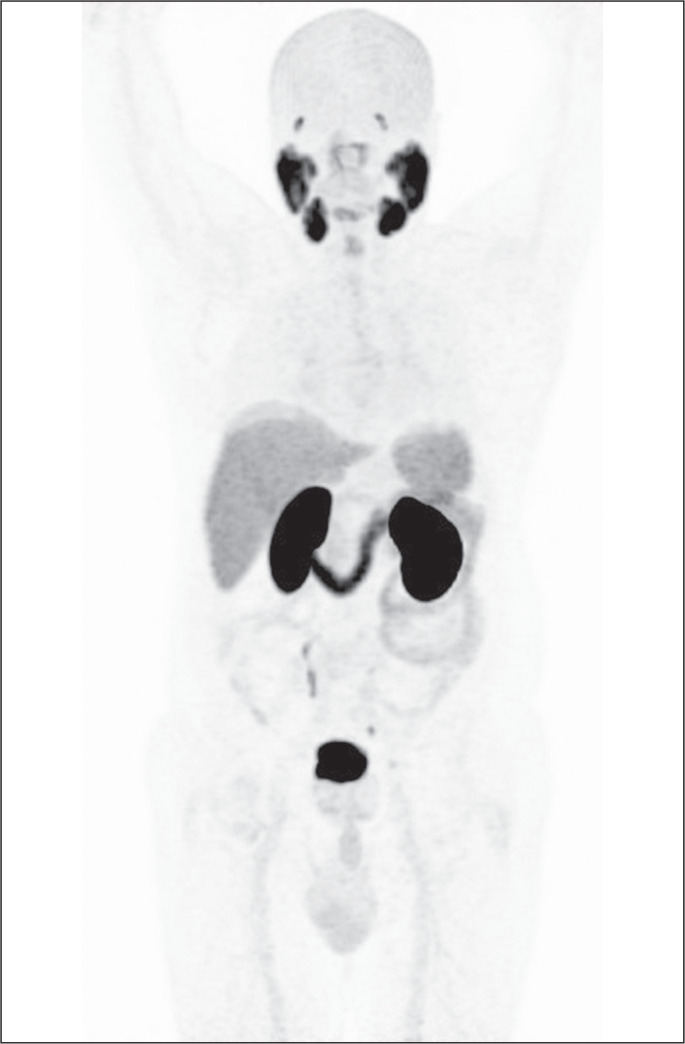
Physiological PSMA uptake in normal tissues. The parotid, submandibular, and lacrimal glands, as well as bladder, kidneys, and small bowel, show intense PSMA uptake. Moderate radiotracer activity is seen in the liver and spleen. The oropharynx, esophagus, larynx, and parasympathetic ganglia can show mild PSMA uptake.

The PSMA radiotracer is taken up in normal tissues. Because PSMA is mainly excreted through the urinary system, the highest-intensity uptake occurs in the kidneys, ureters, and bladder. High physiological PSMA activity is also seen in the lacrimal, parotid, and submandibular glands, whereas the uptake is moderate in the liver and spleen. The parasympathetic ganglia, especially the celiac and stellate ganglia, show faint PSMA uptake. Because PSMA is also excreted in saliva, there might be radiotracer uptake in the oropharynx, esophagus, and larynx. The small bowel, particularly the duodenum, also shows high-intensity PSMA uptake^(2)^, as illustrated in Figure 1.

## MUSCULOSKELETAL CONDITIONS WITH PSMA UPTAKE

Although ^68^Ga-PSMA PET/CT can be used for the detection of bone metastases from prostate cancer, benign musculoskeletal conditions can also show PSMA uptake that can be related to bone remodeling and increased vascularity. Therefore, the correlation with structural imaging methods-CT and magnetic resonance imaging (MRI)-could be important for characterizing the anatomical particularities of such lesions.

### Bone metastasis in prostate cancer

**Figure 2:**
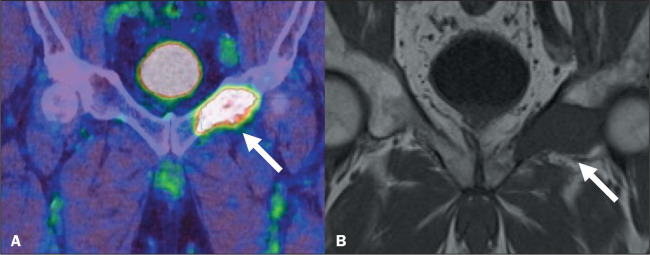
^68^Ga-PSMA PET/CT for prostate cancer staging in a 78-year-old male patient. Abnormal PSMA uptake (SUVmax, 10.4), suggestive of bone metastasis, was identified in the left ischiopubic ramus (arrow in A). Coronal T1-weighted MRI scan (B) showing the corresponding imaging feature (arrow).

The main indication for ^68^Ga-PSMA PET/CT is the staging of high-risk patients and assessment of biochemical recurrence^(1)^. In patients with prostate cancer (Figure 2), greater PSMA expression is associated with higher Gleason scores^(3)^. In addition, because it is effective for imaging disease in lymph nodes, soft tissue, and bone, ^68^Ga-PSMA PET/CT can allow the identification of patients with occult distant metastatic disease^(2)^.

### Fractures

**Figure 3:**
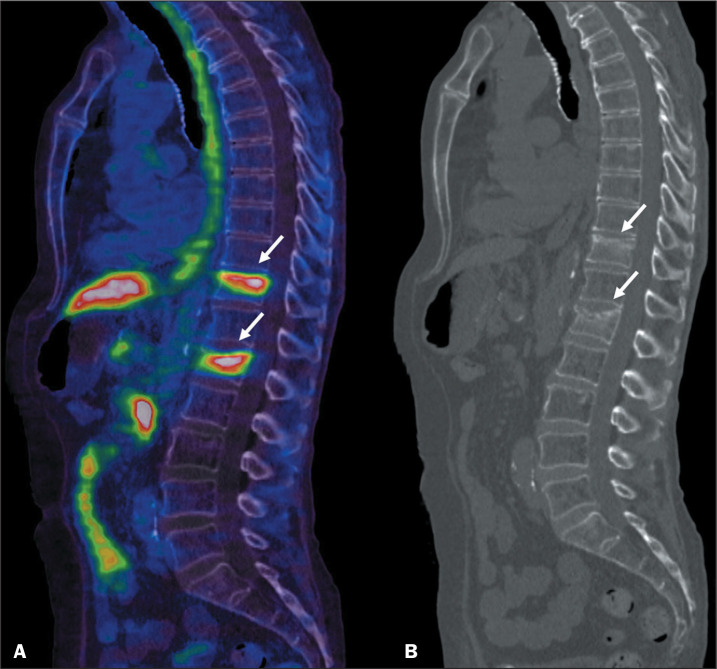
^68^Ga-PSMA PET/CT of an 83-year-old male patient with biochemical recurrence after radical prostatectomy (Gleason score of 4+4). Image A shows abnormal PSMA uptake (arrows) in vertebral bodies T11 and L1 (SUVmax, 6.8 and 6.0, respectively). Sagittal reconstruction of a CT scan (B) showing vertebral compression fractures (arrows).

Fractures in ribs and vertebral bodies, as depicted in Figure 3, have been described as potential pitfalls when reporting ^68^Ga-PSMA PET/CT imaging findings^(4,5)^.

### Degenerative changes

**Figure 4:**
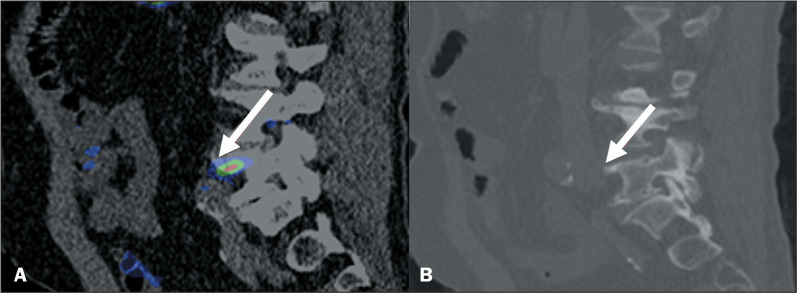
^68^Ga-PSMA PET/CT of a 69-year-old male patient with biochemical recurrence after radical prostatectomy. Abnormal PSMA uptake is seen in vertebral body L5 (arrow in A) (SUVmax, 2.5). Sagittal reconstruction of a CT scan (B) showing an osteophyte (arrow).

Osteodegenerative changes, especially in the spine, can show mild PSMA uptake (Figure 4). The typical imaging findings of osteoarthritis, such as joint space narrowing, subchondral sclerosis, and osteophytes, are diagnostic determinants^(6)^. It has been shown that osteophytes may occasionally present intense PSMA uptake^(7)^.

### Geodes

**Figure 5:**
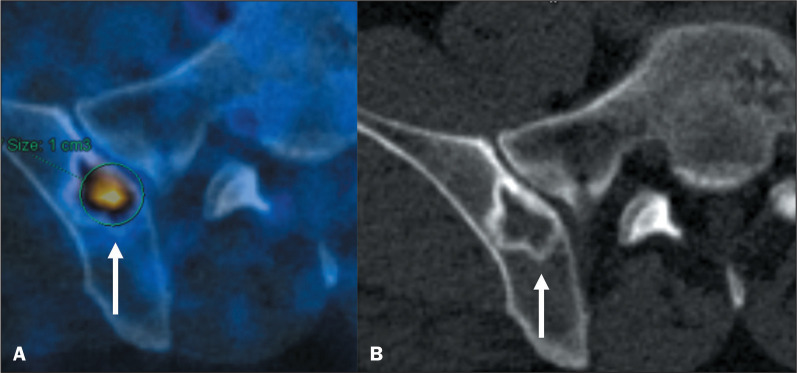
^68^Ga-PSMA PET/CT of a 63-year-old male patient with biochemical recurrence after prostatectomy. Image A shows abnormal PSMA uptake (arrow) in the right iliac bone (SUVmax, 7.2). Coronal reconstruction of a CT scan (B) showing a well-defined lytic lesion with sclerotic margins, characteristic of a geode, in the periarticular surface of the right sacroiliac joint.

Geodes are well-defined lytic lesions in the periarticular space. They are commonly seen in osteodegenerative disease but can also be found in other conditions, such as rheumatoid arthritis and calcium pyrophosphate deposition disease. These lesions may present with mild PSMA uptake^(6)^, as shown in Figure 5.

### Schmorl’s nodes

**Figure 6:**
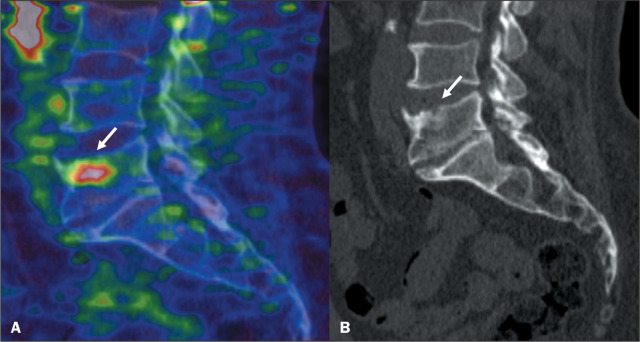
^68^Ga-PSMA PET/CT of a 71-year-old male patient with biochemical recurrence after radical prostatectomy. Image A shows abnormal PSMA uptake (arrow) in vertebral body L5 (SUVmax, 4.3). Sagittal view of a CT scan (B) showing a small nodular lucent lesion with sclerotic margins, consistent with intervertebral disc herniation, on the superior endplate of the lumbar vertebral body (arrow).

Schmorl’s nodes represent intervertebral disc herniation through the cartilaginous and bony endplate into the vertebral body. The main imaging features include a lucent lesion, most commonly in the inferior endplate of the lumbar and lower thoracic vertebrae. Schmorl’s nodes with PSMA uptake can represent a challenge because they mimic bone metastasis (Figure 6). A lack of variation in the imaging findings in comparison with previous examinations can confirm the benign nature of the lesion^(8)^.

### Fibrous cortical defects

**Figure 7:**
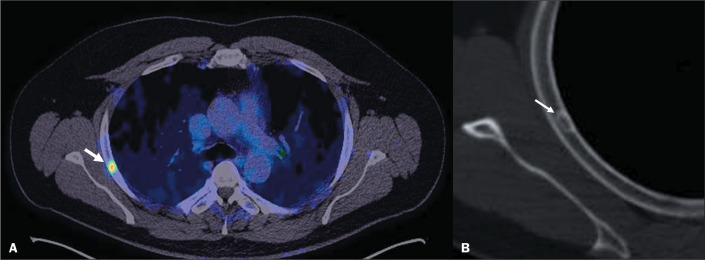
^68^Ga-PSMA PET/CT of a 52-year-old male patient with biochemical recurrence, showing focal uptake (SUVmax, 5.2) in a small hypoattenuating lesion with well delimited sclerotic borders in the lateral segment of the 4th right rib (arrows in A and B). This lesion was comparatively stable in relation to previous studies performed five years before and was therefore characterized as a fibrous cortical defect.

Fibrous cortical defects and non-ossifying fibromas, the latter being a larger lesion (greater than 3 cm), are the most common focal bone lesions^(9)^. These lesions are characterized as lucent lesions with a thin sclerotic rim and no periosteal reaction. In some cases, there is mild PSMA uptake in the ribs, which can be associated with a small fibrous cortical defect^(6)^, as illustrated in Figure 7.

### Fibrous dysplasia

**Figure 8:**
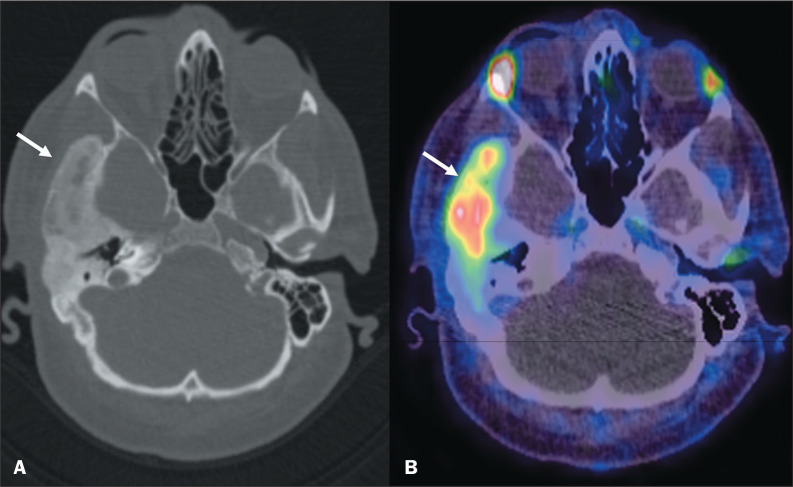
Preoperative ^68^Ga-PSMA PET/CT of a 58-year-old male patient, performed for the staging of prostate cancer. CT scan (A) showing increased bone thickness with homogeneous ground-glass opacity and loss of the normal trabecular pattern, suggestive of fibrous dysplasia, in the right temporal bone (arrow). Note the PSMA uptake (arrow) in B (SUVmax, 5.9).

Fibrous dysplasia is a developmental anomaly in which normal bone is replaced by poorly organized fibrous tissue. It can be monostotic (involving only one bone) or polyostotic (involving multiple bones) and has varied imaging manifestations. The typical radiological feature is an expansile, well-circumscribed, homogenous lesion with a ground-glass appearance^(10)^. There have been reports of moderate PSMA uptake in such lesions^(11)^, as depicted in Figure 8.

### Paget’s disease

**Figure 9:**
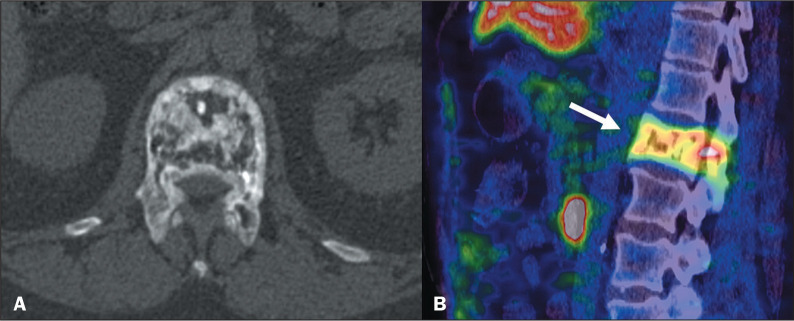
Preoperative ^68^Ga-PSMA PET/CT of a 70-year-old male patient, performed for the staging of prostate cancer, showing coarse trabecular thickening in vertebral body L1 (A), together with cortical sclerosis and thickening, representing the picture frame sign, imaging findings typical of Paget’s disease. Note also the high tracer uptake (arrow) in B (SUVmax, 25.4).

Paget’s disease is a chronic skeletal disorder characterized by excessive osseous remodeling. Abnormal resorption and apposition of bone creates varying clinical and radiologic manifestations^(12)^. Paget’s disease has been described as a potential mimicker of bone metastases on ^68^Ga-PSMA PET/CT and usually presents with low to moderate radiotracer uptake^(13)^, as shown in Figure 9. It is thought that the angiogenesis induced by Paget’s disease is the underlying mechanism: the endothelia of those vessels express PSMA receptors.

### Vertebral hemangiomas

**Figure 10:**
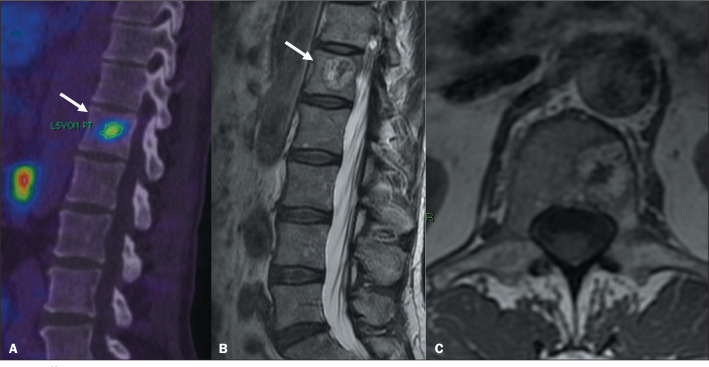
^68^Ga-PSMA PET/CT of a 67-year-old patient with biochemical recurrence after radical prostatectomy. Image A shows abnormal PSMA uptake (arrows) in vertebral body L1 (SUVmax, 8.5). The lesion was hyperintense on sagittal T2-weighted and axial T1 weighted sequences (B and C, respectively), demonstrating high fat content, consistent with a vertebral hemangioma.

Vertebral hemangiomas are common benign vascular tumors that appear in the spine. The typical appearance on CT is thickened vertebral trabeculae, whereas MRI, in typical hemangiomas, shows the fat content (high signal intensity on T1-weighted sequences) and the water content (high signal intensity on T2-weighted sequences). These benign tumors have been reported to mimic prostate cancer because of their PSMA uptake^(14)^, as portrayed in Figure 10. The PSMA uptake of such tumors is variable, and those with higher uptake present PSMA expression in their endothelial cells^(6)^.

### Bursitis

**Figure 11:**
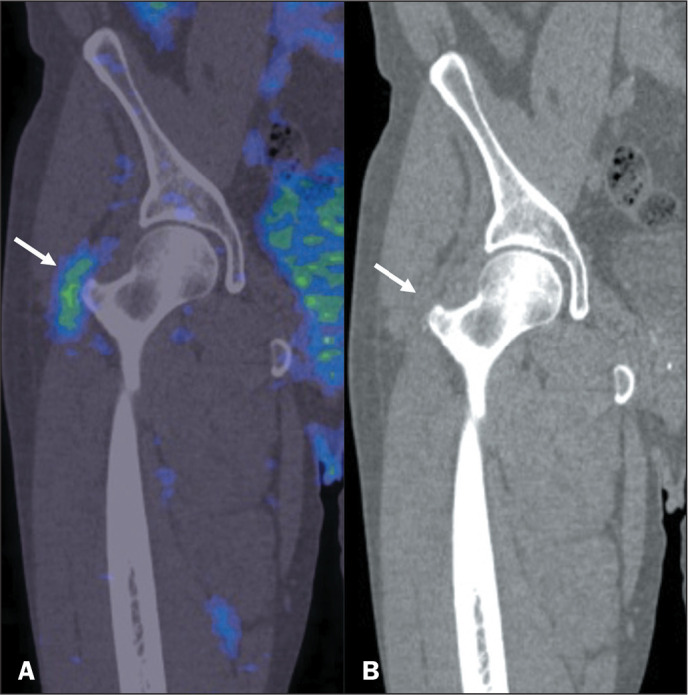
^68^Ga-PSMA PET/CT of a 60-year-old male patient, performed for the staging of prostate cancer, showing abnormal PSMA uptake (SUVmax, 2.5) in the pertrochanteric region (arrow in A). Coronal view of a CT scan (B) showing a low-density area in the trochanteric bursa topography, corresponding to trochanteric bursitis.

It has been reported that PSMA uptake occurs in various inflammatory conditions, including bursitis (Figure 11), which is an inflammatory condition of the bursa^(15)^.

## CONCLUSION

Benign bone and soft-tissue lesions can mimic malignancy, particularly if they are highly avid for ^68^Ga-PSMA. Musculoskeletal pitfalls can be avoided as the radiologist becomes familiar with their appearance, understands their causes, and fully analyzes their morphologic features on unfused CT/MRI images.
